# Species composition and diversity of ground bryophytes across a forest edge-to-interior gradient

**DOI:** 10.1038/s41598-018-30400-1

**Published:** 2018-08-08

**Authors:** Tiantian Jiang, Xuecheng Yang, Yonglin Zhong, Qiming Tang, Ying Liu, Zhiyao Su

**Affiliations:** College of Forestry and Landscape Architecture, South China Agricultural University, Guangzhou, 510642 China

## Abstract

Understanding diversity patterns and community structure of bryophytes will help integrate nature conservation at multiple biotic-group levels. We conducted a survey of ground bryophytes in a subtropical forest along an edge-to-interior gradient in South China. We recorded 11 liverwort species from 10 genera of seven families, and 26 moss species from 23 genera of 16 families in three transects. A two-way cluster analysis detected the environmental gradient between the forest edge and forest interior for bryophytes with habitat specificity. Functional diversity of bryophytes differed significantly across an edge-to-interior gradient. The range and median in both structural and functional diversity decreased remarkably from the forest edge to the interior. Multi-response permutation procedures showed significant differences in species composition between the forest-edge and forest-interior, and between the intermediate and forest-interior transects. Seven species were detected with a significant indicator value for indicating environmental conditions in the forest edge, while only one such species was found indicative of the intermediate transect. Our results demonstrate that remarkable edge effects exist for species composition and functional diversity patterns, and the forest edge is a marginal habitat with high biotic heterogeneity. Furthermore, functional diversity metrics are more sensitive to the edge effect than species diversity.

## Introduction

Bryophytes, known as liverworts, mosses, and hornworts, are the earliest land plants in the phylogenetic systematics of the plant kingdom^1^. They occur widely in the global terrestrial ecosystem, often as dominants in the floor layer of the moist tropical and subtropical broadleaved forest biomes^2–4^. However, bryophytes appear to have been neglected in many ecological studies, where only vascular plants or even woody plants were investigated such as in the emerging fields of community ecology to explore the role of ecological processes and biotic diversity in maintaining ecosystem function^5,6^. Until now, little has been known about the diversity patterns of bryophytes, their spatial heterogeneity, their role in forest community assembly, and their biotic and abiotic interactions to maintain the ecosystem function as a whole. The knowledge gap surrounding bryophyte community function is even greater for tropical and subtropical forest ecosystems, thus hampering our steps towards a better understanding of the ecosystem functions as a whole.

Botanists have argued that as the earliest land plants, bryophytes reflect the dispersal history of plants of various evolutionary stages in the terrestrial ecosystem^1,7^, and their physiological adaptation, community structure, and the ecological functions in response to environmental change are much more complicated than previously imagined^8^. Disseminated by spores, bryophytes have an outstanding capability for dispersal and will respond sensitively to environmental change. Previous studies have demonstrated that bryophytes are good bio-indicators for environmental pollution due to the special leaf architecture of the plant organism^9–12^. However, to gain a whole picture of the bryophytes’ distribution, diversity patterns, community structure, and their response to ecological factors along environmental gradients, extensive studies in the community ecology of bryophytes should be carried out in natural ecosystems, especially in the forest ecosystem.

As an essential property of the forest ecosystem, species diversity and the functional diversity of a community reflect the biotic response to habitat heterogeneity and are the result of biotic and abiotic interaction^13,14^. A comprehensive knowledge of the diversity and its spatial patterns at various levels of biological groups is crucial for both regional and local biodiversity conservation planning, and will provide insights into further exploration of the relationships between organisms and their environments. Currently, many bryophyte studies are focused on the botanical and floristic aspects, particularly for the compilation of floristic inventory and plant checklists^15,16^. A few studies have revealed the effect of various disturbance regimes on bryophyte diversity^11,17^; other investigations have analyzed the response of bryophytes to precipitation, acid deposition, and topographic factors^9,18,19^. In the vast areas of China, the limited research reports on bryophytes have concentrated on the Changbai Mountain Range^20^, Qilian Mountain Range^12^, and regions such as the Yunnan-Guizhou Plateau^19^. Very rarely have studies reported on the ecology of bryophytes distributed in South China, especially in the subtropical area of Guangdong province^18^.

Edge effect is one of the generally-recognized mechanisms driving plant diversity in forest ecosystems^21–23^ and has significant implications for forest management and habitat conservation. The effect arising from a forest edge represents the interaction of biotic and abiotic factors rather than a single site factor^24,25^. Although edge effect is a widespread ecological phenomenon and has been explored for vascular plant diversity^26,27^, few studies have investigated this for bryophytes^28–35^. In this study, we conducted a survey of the understory ground bryophytes using a quadrat sampling method in a subtropical broadleaved forest along an edge-to-interior gradient, followed by multivariate statistical analysis of the field data. We aimed to address the following questions: (1) Do bryophyte assemblages show significant species-specific associations across an edge-to-interior gradient? (2) How are bryophyte species composition, community structure, and diversity related to the edge-to-interior gradient? and (3) Are there species with high habitat specificity that can act as bio-indicators for significant species-habitat association?

## Results

### Species Composition and Community Structure

A total of 37 ground bryophyte species from 33 genera of 23 families were recorded from the forest understory within the 2-ha plot. Out of the total number, 11 species from 10 genera of 7 families were liverworts, while 26 species from 23 genera of 16 families were mosses (Table 1). No hornworts were found. High ecological dominance existed in the bryophyte community. The moss species *Pseudotaxiphyllum pohliaecarpum* was the most dominant species, with an importance value (IV) = 25.99, relative frequency (RF) = 15.93, and relative cover (RC) = 10.06. Six other bryophyte species with an IV ≥ 10 found to be dominant were as follows: *Kurzia gonyotricha*, *Bazzania tridens*, *Chiloscyphus latifolius*, *Leucobryum juniperoideum*, *Fissidens laxus*, *Haplocladium microphyllum*. In contrast, eight bryophyte species, with an IV < 1, were found to have occurred in only one sample unit. The lowest IV (0.35) belonged to *Pallavicinia lyellii*, and seven other such bryophyte species were as follows: *Chiloscyphus profundus*, *Campylopus atroviren*, *Homaliodendron flabellatum*, *Macromitrium schmidii* var. *macroperichaetialium*, *Pogonatum inflexum*, *Fauriella tenuis*, *Neckeropsis calcicola* (Table 1).

**Table 1:** Taxonomic composition of bryophytes and community structural attributes.

Family	Species	F	AC	RF	RC	IV
**Liverworts**						
Lepidoziaceae	*Bazzania tridens*	23	28.69	7.80	6.93	14.72
Lophocoleaceae	*Chiloscyphus latifolius*	28	15.62	9.49	3.77	13.26
Lophocoleaceae	*Chiloscyphus profundus*	1	0.10	0.34	0.02	0.36
Lophocoleaceae	*Heteroscyphus zolliingeri*	3	15.30	1.02	3.69	4.71
Lepidoziaceae	*Kurzia gonyotricha*	33	39.98	11.19	9.65	20.84
Lejeuneaceae	*Lejeunea eifrigii*	8	5.94	2.71	1.43	4.15
Metzgeriaceae	*Metzgeria conjugata*	5	5.65	1.69	1.36	3.06
Pallaviciniaceae	*Pallavicinia lyellii*	1	0.05	0.34	0.01	0.35
Plagiochilaceae	*Plagiochila flexuosa*	4	12.30	1.36	2.97	4.33
Radulaceae	*Radula obscura*	3	9.80	1.02	2.37	3.38
Lejeuneaceae	*Spruceanthus polymorphus*	4	3.30	1.36	0.80	2.15
**Mosses**						
Meteoriaceae	*Aerobryopsis wallichii*	5	6.40	1.69	1.54	3.24
Pylaisiadelphaceae	*Brotherella henonii*	10	6.51	3.39	1.57	4.96
Leucobryaceae	*Campylopus atrovirens*	1	0.20	0.34	0.05	0.39
Hypnaceae	*Ectropothecium dealbatum*	6	13.00	2.03	3.14	5.17
Entodontaceae	*Entodon schleicheri*	5	7.78	1.69	1.88	3.57
Heterocladiaceae	*Fauriella tenuis*	1	0.40	0.34	0.10	0.44
Fissidentaceae	*Fissidens laxus*	25	13.72	8.47	3.31	11.79
Fissidentaceae	*Fissidens nobilis*	2	2.53	0.68	0.61	1.29
Fissidentaceae	*Fissidens oblongifolius*	8	17.89	2.71	4.32	7.03
Thuidiaceae	*Haplocladium microphyllum*	9	30.15	3.05	7.28	10.33
Anomodontaceae	*Herpetineuron toccoae*	5	23.40	1.69	5.65	7.34
Neckeraceae	*Homalia trichomanoides*	3	0.90	1.02	0.22	1.23
Neckeraceae	*Homalia trichomanoides var*. *japonica*	8	9.43	2.71	2.28	4.99
Neckeraceae	*Homaliodendron flabellatum*	1	0.20	0.34	0.05	0.39
Hypnaceae	*Hypnum fauriei*	3	8.20	1.02	1.98	3.00
Leucobryaceae	*Leucobryum juniperoideum*	14	32.65	4.75	7.88	12.63
Orthotrichaceae	*Macromitrium schmidii var*. *macroperichaetialium*	1	0.20	0.34	0.05	0.39
Neckeraceae	*Neckeropsis calcicola*	1	1.80	0.34	0.43	0.77
Mniaceae	*Plagiomnium rhynchophorum*	1	3.60	0.34	0.87	1.21
Polytrichaceae	*Pogonatum inflexum*	1	0.30	0.34	0.07	0.41
Hypnaceae	*Pseudotaxiphyllum pohliaecarpum*	47	41.68	15.93	10.06	25.99
Brachytheciaceae	*Rhynchostegium pallidifolium*	3	10.50	1.02	2.53	3.55
Sematophyllaceae	*Sematophyllum subpinnatum*	2	9.00	0.68	2.17	2.85
Calymperaceae	*Syrrhopodon prolifer*	3	1.90	1.02	0.46	1.48
Hypnaceae	*Taxiphyllum taxirameum*	5	12.90	1.69	3.11	4.81
Thuidiaceae	*Thuidium pristocalyx*	12	22.30	4.07	5.38	9.45

Abbreviations: F = frequency; AC = average cover; RF = relative frequency; RC = relative cover; IV = importance value.

### Bryophyte Distribution and Interspecific Association

**Figure 1:**
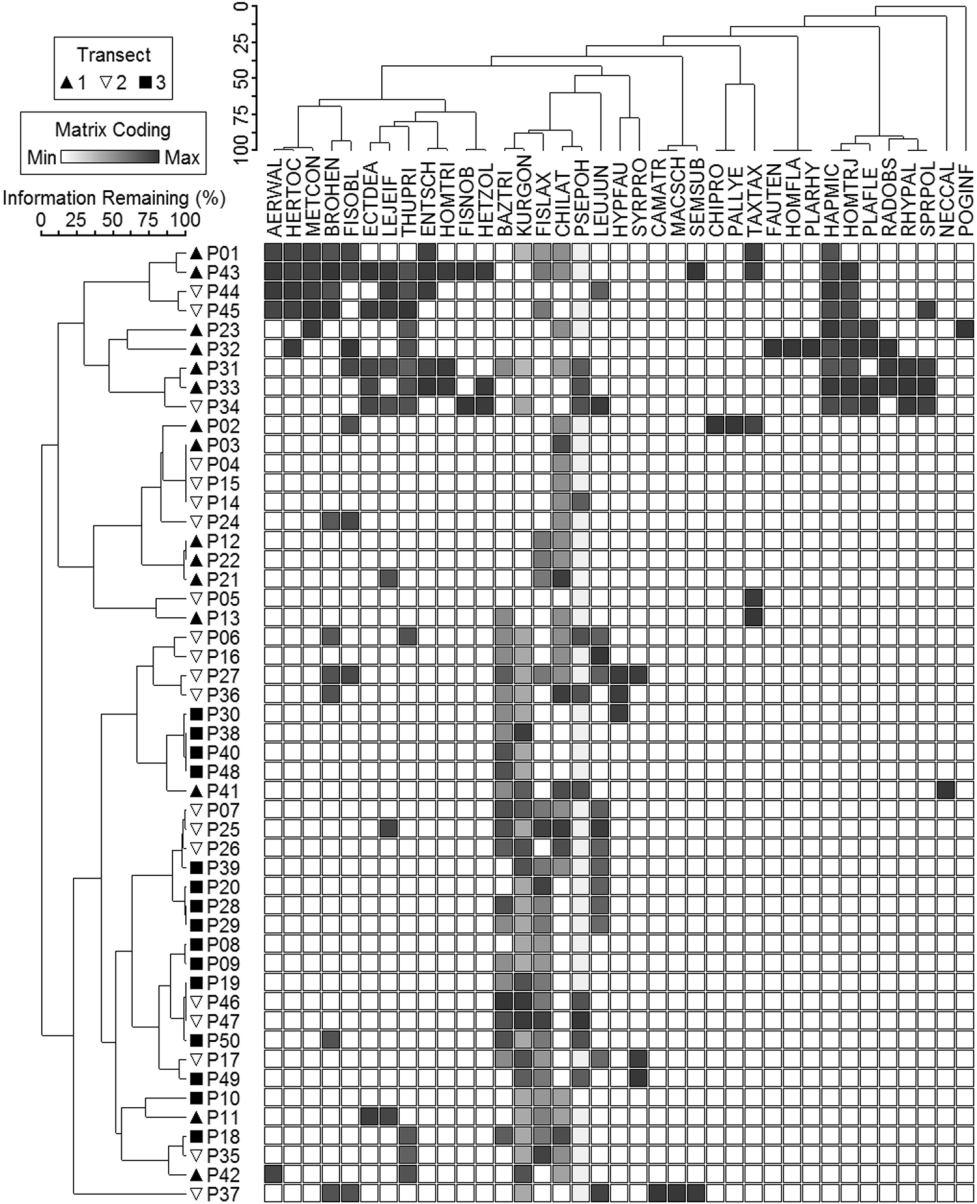
Two-way cluster dendrogram showing plot groupings, and bryophyte species composition and distribution. Each plot is symbol coded for its membership to a particular transect. The grey scale for the Matrix Coding from light to dark corresponds to cover class value in an increasing order. Species code: AERWAL = *Aerobryopsis wallichii*; BAZTRI = *Bazzania tridens*; BROHEN = *Brotherella henonii*; CAMATR = *Campylopus atrovirens*; CHILAT = *Chiloscyphus latifolius*; CHIPRO = *Chiloscyphus profundus*; ECTDEA = *Ectropothecium dealbatum*; ENTSCH = *Entodon chleicheri*; FAUTEN = *Fauriella tenuis*; FISLAX = *Fissidens laxus*; FISNOB = *Fissidens nobilis*; FISOBL = *Fissidens oblongifolius*; HAPMIC = *Haplocladium microphyllum*; HERTOC = *Herpetineuron toccoae*; HETZOL = *Heteroscyphus zolliingeri*; HOMFLA = *Homaliodendron flabellatum*; HOMTRI = *Homalia trichomanoides*; HOMTRJ = *Homalia trichomanoides* var. *japonica*; HYPFAU = *Hypnum fauriei*; KURGON = *Kurzia gonyotricha*; LEJEIF = *Lejeunea eifrigii*; LEUJUN = *Leucobryum juniperoideum*; MACSCH = *Macromitrium schmidii* var. *macroperichaetialium*; METCON = *Metzgeria conjugata*; NECCAL = *Neckeropsis calcicola*; PALLYE = *Pallavicinia lyellii*; PLAFLE = *Plagiochila flexuosa*; PLARHY = *Plagiomnium rhynchophorum*; POGINF = *Pogonatum inflexum*; PSEPOH = *Pseudotaxiphyllum pohliaecarpum*; RADOBS = *Radula obscura*; RHYPAL = *Rhynchostegium pallidifolium*; SEMSUB = *Sematophyllum subpinnatum*; SPRPOL = *Spruceanthus polymorphus*; SYRPRO = *Syrrhopodon prolifer*; TAXTAX = *Taxiphyllum taxirameum*; THUPRI = *Thuidium pristocalyx*.

The distribution of bryophyte species in response to the edge-to-interior gradient is visualized by a two-way cluster dendrogram (Fig. 1). The clustering of sample units clearly separated the forest-edge transect from the forest-interior transect. Sample units in the intermediate transect were not distributed in one cluster, instead they were dispersed in either the edge or interior transects, indicating their intermediate nature in habitat conditions as the transition between the forest edge and interior transects. With regard to species groupings, contrasting distribution patterns were found in both common species with coincidence in a number of sample units, representing high interspecific association, and in the rare species unique to only one or two sample units. For example, *Pseudotaxiphyllum pohliaecarpum* and *Chiloscyphus latifolius* occurred in almost all the sample units, illustrating their adaptability to heterogeneous habitats, while *Fissidens laxus*, *Kurzia gonyotricha*, and *Bazzania tridens* only dominated the intermediate and the forest-interior transects, indicating that the forest-edge habitat might act as an ecological filter for their distribution. Nine bryophyte species were detected as rare species with single occurrence in the sample units: *Campylopus atrovirens*, *Chiloscyphus profundus*, *Fauriella tenuis*, *Homaliodendron flabellatum*, *Macromitrium schmidii* var. *macroperichaetialium*, *Neckeropsis calcicola*, *Pallavicinia lyellii*, *Plagiomnium rhynchophorum*, and *Pogonatum inflexum*. Some of these species occurred with coincidence in the same sample unit (Fig. 1), showing high interspecific association.

### Species Diversity and Functional Diversity

**Figure 2:**
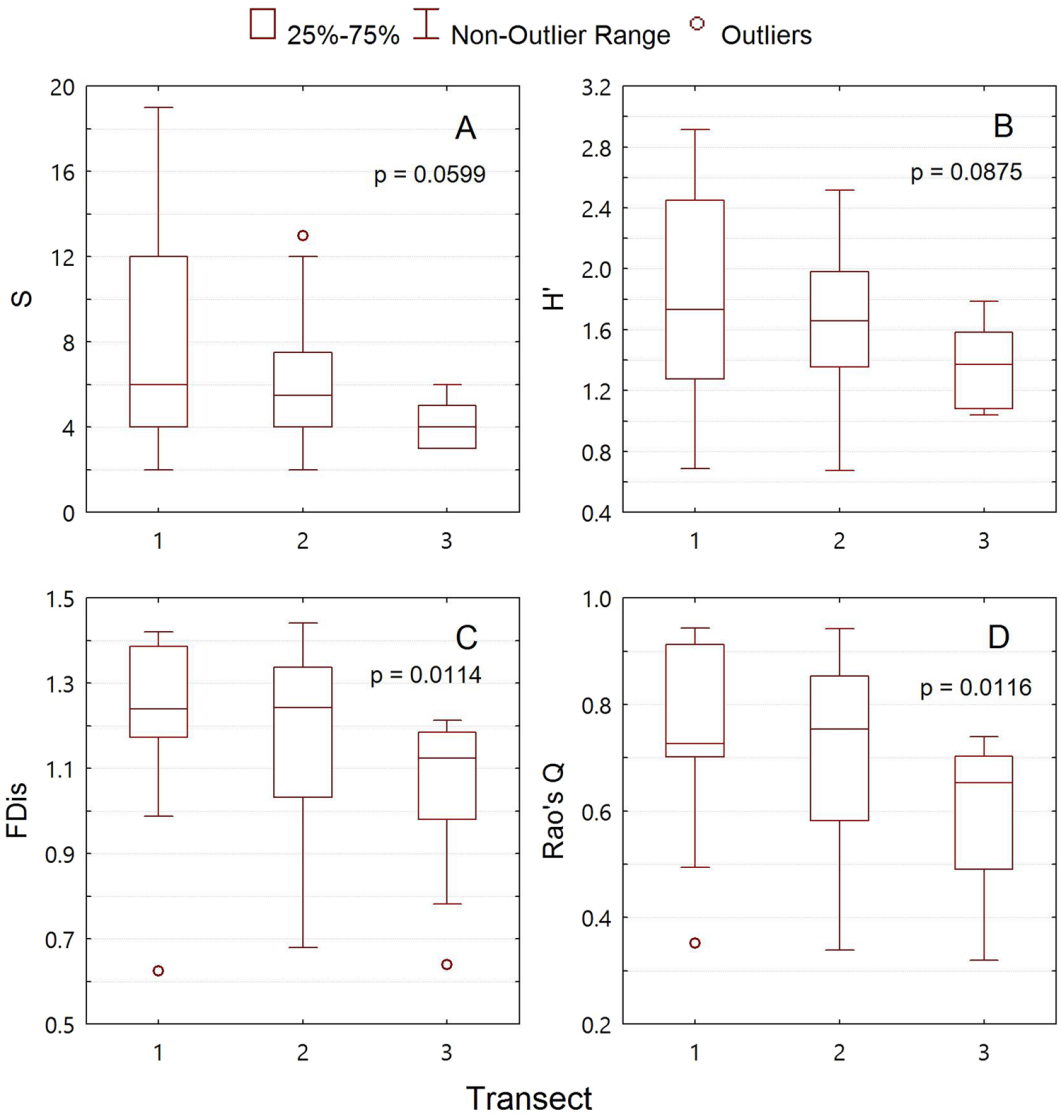
Changes in bryophyte structural and functional diversity across transects. *S* is the species richness; *H′* is the Shannon-Wiener diversity index; FD is the functional dispersion; and Rao’s Q is Rao’s quadratic entropy. Transect code: 1 = Forest-edge transect; 2 = Intermediate transect; 3 = Forest-interior transect.

**Figure 3:**
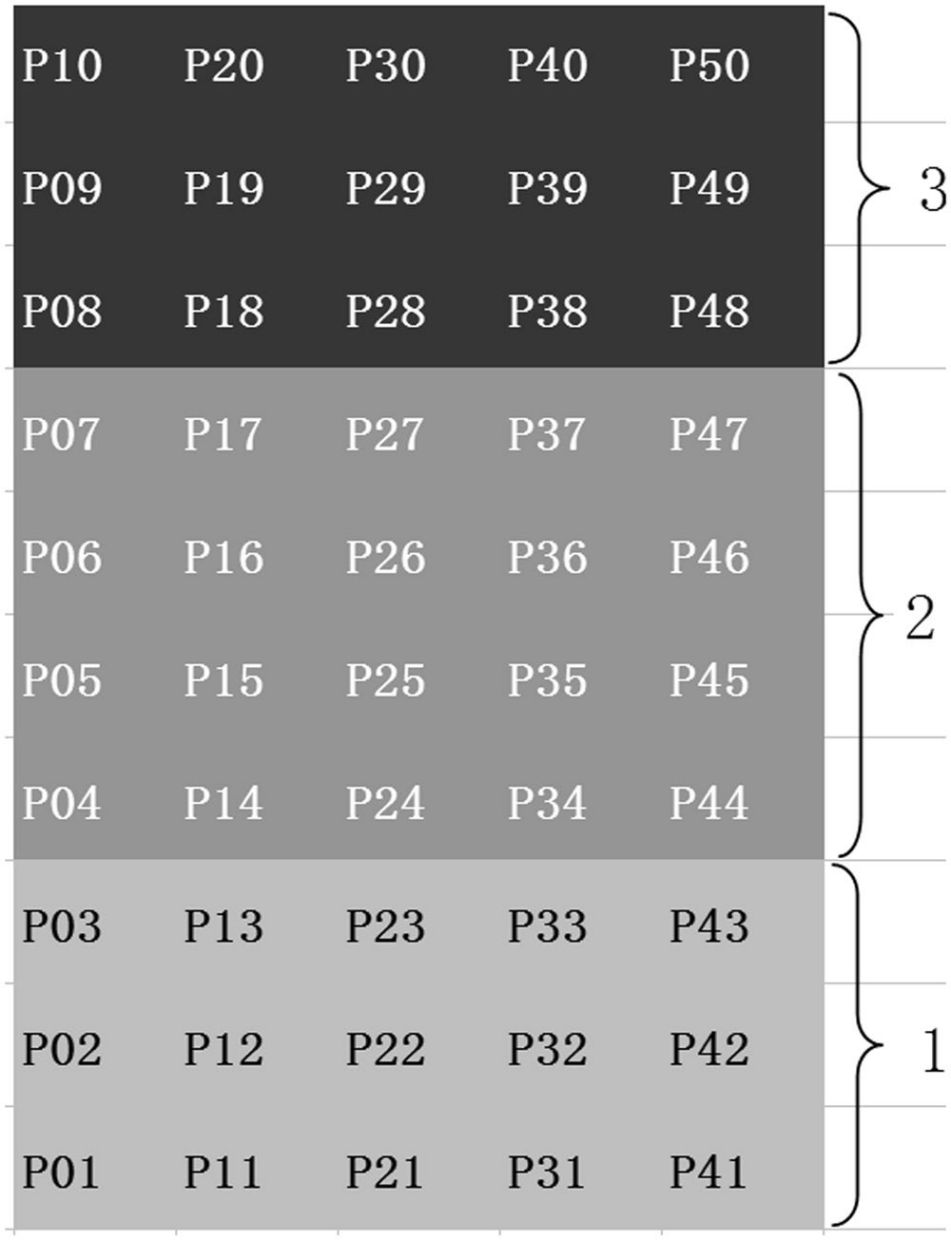
Plot layout for the sampling of understory ground bryophytes and the habitat gradient by transect. Transect code: 1 = Forest-edge transect; 2 = Intermediate transect; 3 = Forest-interior transect.

Changes in both species diversity and functional diversity showed a decreasing trend across an edge-to-interior gradient (Fig. 2). However, no significant difference in species richness (Fig. 2A, p = 0.599) and Shannon-Wiener diversity index (Fig. 2B, p = 0.0875) were found across the forest-edge, intermediate, and forest-interior transects, whereas significant edge effects on functional diversity were exhibited in the two functional diversity metrics, FDis (Fig. 2C, p = 0.0114) and Rao’s Q (Fig. 2D, p = 0.0116). These gradients were reflected in the median and the min-max range differences of the structural and functional diversity metrics between the forest-interior and the intermediate transects, or between the forest-interior and the forest-edge transects. The range in both species richness and Shannon-Wiener diversity index remarkably decreased from the forest-edge to the forest-interior transects, with the lowest range and median found in the forest-interior (Fig. 2A,B), while for functional diversity, high ranges were found in the forest-edge as well as the intermediate transects (Fig. 3C,D).

### Indicator Species

Multi-response permutation procedures (MRPP) showed an extremely significant difference in species composition across an edge-to-interior gradient in an overall comparison (Table 2, P < 10^−6^). The MRPP pairwise comparison detected extremely significant differences between the forest-edge and forest-interior transects (Table 2, P < 10^−6^), and between the intermediate and forest-interior transects (Table 2, P < 10^−4^). The results from MRPP indicated that both the forest-edge and forest-interior transects had high habitat specificity for the bryophyte species composition and distribution.

**Table 2:** Multi-response Permutation Procedure (MRPP) for bryophyte species composition among the transects.

Transects for comparison	Variance	Skewness	*T*	*A*	*P*
Overall comparison	0.399	−0.946	−8.731	0.090	<10^−6^
Pairwise comparison					
1 versus 2			−1.813	0.019	0.058
1 versus 3			−11.799	0.145	<10^−6^
2 versus 3			−6.602	0.067	<10^−4^

*A* represents the “effect size” of within-group homogeneity as compared to the random expectation; *T* is a statistic describing the separation between the groups; and *P* is the *P*-value from the significance test of homogeneity. Transect code: 1 = Forest-edge transect; 2 = Intermediate transect; 3 = Forest-interior transect.

Seventeen species had an indicator value >10 for indicating a particular transect habitat, but only eight indicator species were detected to have a significant indicator value (Table 3). Seven such species with a significant indicator value were found confined to the forest edge. These were *Haplocladium microphyllum*, *Fissidens oblongifolius*, *Homalia trichomanoides* var. *japonica*, *Entodon schleicheri*, *Taxiphyllum taxirameum*, *Homalia trichomanoides*, and *Radula obscura*. Only one species, *Leucobryum juniperoideum*, was significantly indicative to the intermediate transect with the maximum indicator value.

**Table 3:** Indicator species of bryophytes with an indicator value >10 across transects.

Species	Transect	Observed indicator value	Indicator value from randomization		*p*
			Mean	Standard deviation	
*Leucobryum juniperoideum*	Intermediate	37	17.4	5.45	0.007
*Fissidens laxus*	Forest-interior	32.9	24.9	5.08	0.078
*Haplocladium microphyllum*	Forest-edge	28.4	13.3	5.1	0.015
*Fissidens oblongifolius*	Forest-edge	25.4	12.9	5.38	0.031
*Homalia trichomanoides var*. *japonica*	Forest-edge	24.5	12.5	4.98	0.022
*Entodon schleicheri*	Forest-edge	22.7	9.9	4.77	0.036
*Thuidium pristocalyx*	Forest-edge	21.8	15.8	5.26	0.129
*Taxiphyllum taxirameum*	Forest-edge	21.3	9.9	4.72	0.042
*Homalia trichomanoides*	Forest-edge	20	7.7	4.27	0.046
*Radula obscura*	Forest-edge	20	7.7	4.24	0.047
*Brotherella henonii* var. *henonii*	Intermediate	20	14.2	5.12	0.121
*Ectropothecium dealbatum*	Forest-edge	19.9	10.7	4.81	0.063
*Plagiochila flexuosa*	Forest-edge	16.2	8.8	4.27	0.069
*Lejeunea eifrigii*	Forest-edge	13.6	12.7	5.11	0.369
*Aerobryopsis wallichii*	Forest-edge	13	9.7	4.59	0.249
*Herpetineuron toccoae*	Forest-edge	12.6	9.8	4.58	0.273
*Metzgeria conjugata*	Forest-edge	12.3	9.8	4.66	0.284

## Discussion

Bryophyte diversity patterns and community structure changed in response to environmental gradients. Except for the intermediate transect, high habitat specificity for the bryophyte species was found in both the forest-edge and forest-interior transects. High interspecific association existed in both common species that had co-occurrence in a number of sample units, and rare species which were found in only one or two sample units. Two bryophyte species, *Pseudotaxiphyllum pohliaecarpus*, and *Chiloscyphus latifolius*, occurred in almost all sample units, reflecting their robust adaptability to heterogeneous habitats. Although three common species, *Fissidens laxus*, *Kurzia gonyotricha*, and *Bazzania tridens*, were restricted to the intermediate and the forest-interior transects, they occurred in most sample units of the two transects, indicating that the forest-edge habitat might act as an environmental filter for their distribution. The nine bryophyte species detected as rare species with unique occurrence in the sample units demonstrated high habitat specificity, and a very high species association was exhibited in these species with coincidence in the same sample unit.

Biodiversity is regarded as the essential property characteristic of an ecosystem or biotic community^36–38^. In our study, changes of species richness, Shannon-Wiener diversity, and functional diversity showed similar decreasing trends from the forest edge to the forest interior, and the functional diversity exhibited a significant edge effect. The range in both species richness and Shannon-Wiener diversity index decreased remarkably from the forest-edge to the forest-interior transects, with the lowest range and median found in the forest-interior. These differences were reflected in the discrepancy between the forest-interior and the intermediate transects, or between the forest-interior and the forest-edge transects. Previous studies have demonstrated that changes to understory microclimate are responsible for the differences in forest interior vs. edge environments^29,39–42^, but in our study, the influence by the typhoons occurring in the South China sea from July to November each year may be the major cause for the edge-interior gradient^43,44^. The highest heterogeneity of the forest-edge transect for bryophyte diversity may have arisen from the effect of wind from various directions in a year, which serves as the major medium for the dispersal and colonization of bryophyte propagules^45–47^. In the forest-interior transect, mitigation of the wind effect by the obstruction of tree trunks and crown foliage may have led to the lower variability in species diversity. Moreover, significant edge effect was found for functional diversity, but not for species diversity in the forest edge when compared to the forest interior. This indicated that the functional diversity metrics were more sensitive than species diversity metrics in characterizing the forest edge-to-interior gradient.

The forest edge-to-interior gradient for bryophyte species composition and diversity patterns were also corroborated by multi-response permutation procedures (MRPP). The MRPP showed an extremely significant difference in species composition across an edge-to-interior gradient by overall comparison (*P* < 10^−6^), and by pairwise comparison between the forest edge vs. forest interior transects and between the forest intermediate vs. forest interior transects (*P* < 10^−4^). The results from the MRPP indicated that both the forest-edge and forest-interior transects had high habitat specificity for bryophyte species composition and distribution. Further evidence for habitat specificity of the forest-edge transect was provided by indicator species analysis (ISA). Eight species (*Leucobryum juniperoideum*, *Haplocladium microphyllum*, *Fissidens oblongifolius*, *Homalia trichomanoides* var. *japonica*, *Entodon schleicheri*, *Taxiphyllum taxirameum*, *Homalia trichomanoides*, and *Radula obscura*) were detected to have a significant indicator value. Seven such species with a significant indicator value were found with a restricted occurrence in the forest edge, while only one species (*Leucobryum juniperoideum*) was significantly indicative of the intermediate transect with the maximum indicator value. The significant indicator species were the representations of both species habitat association and habitat specificity.

## Conclusions

In conclusion, the spatial patterns of bryophyte species diversity and community structure in response to the edge-to-interior gradient were determined by habitat characteristics as well as the bryophytes’ biological properties. Our results demonstrated that the conspicuous diversity patterns, community structure, and interspecific association in ground bryophytes are related to the edge-to-interior gradient, and the forest edge is a special habitat with high biotic heterogeneity. Further studies are required to explore issues such as the drivers of the spatial patterns in bryophyte distribution, the mechanisms for the maintenance of bryophyte diversity, and to what extent edge effects influence bryophyte species diversity and functional diversity patterns under climate change.

## Materials and Methods

### Study Area

We conducted a field survey to collect bryophyte vegetation data in the Kanghe Provincial Nature Reserve (115°04′–115°09′E, 23°44′–23°53′N). This nature reserve is located in the southeastern part of South China’s Guangdong province, approximately 220 km from Guangzhou, the capital city of Guangdong province. The terrain of the nature reserve is hilly and mountainous, with the highest peak at 839.7 m a.s.l. The area has a subtropical monsoon climate, with a mean annual precipitation of 2142 mm, a mean annual temperature ranging from 20.3–21.1 °C, and a frost-free period of 345–350 d^43,48^. From July to November each year, this area is regularly affected by typhoons, the tropical cyclones occurring in the South China sea^43,49^. The soil type is a clay loamy latosolic red soil with a thick soil layer. Vegetation in the area is dominated by subtropical evergreen broadleaved forests. According to previous studies^48,50^, the forest canopy is dominated by a number of hardwood tree species such as *Castanopsis carlesii*, *Schima superba*, *Castanopsis fargesii*, and *Itea chinensis*.

### Sampling Design and Bryophyte Census

A 2-ha plot was delineated within a relatively homogeneous broadleaved forest. The plot was further divided into a grid system of fifty 20 × 20 m subplots, coded as P01–P50 using a total station (Nikon DTM 310). These subplots were grouped into three transects, i.e., the forest-edge transect, the intermediate transect, and the forest-interior transect (Fig. 3), according to their locations relative to the forest edge, which is adjacent to an open, non-forested area, with a major forest path passing by. The average elevation of the 2-ha plot is 235 m. Five 2 × 2 m quadrats were laid out at positions along two diagonal lines of each 400-m^2^ subplot, one at the intersecting point (the central location of the subplot), and two at the 1/4 and 3/4 points of each of the two diagonal lines. For easy and accurate estimation of percent cover, bryophytes in each quadrat were censused by four 1-m^2^ sub-quadrats in an anticlockwise sequence for species identity, percent cover, and habitat attributes. Voucher specimens of the bryophytes were collected, tagged, and placed in kraft paper envelopes for identification in the laboratory using a microscope. Field sampling was conducted from September to October, 2016. Bryophyte systematics and nomenclature followed the *Bryophyte Flora of Guangdong*^51^.

### Structural and Functional Diversity Indexes

All field data were pooled into the subplot level to compute relative frequency, relative cover, and importance value by species, species richness, the Shannon-Wiener diversity index, and functional diversity by subplots. We used percent cover data directly to calculate importance value, but for the calculation of diversity metrics and the construction of a plot × species dataset for multivariate analysis, we first transformed percent cover data into cover class and then used the median of the cover class code by subplots in the analysis (Supplementary Table S1). Bryophyte plants are tiny and usually grow in short turfs or wefts, so it is not easy to estimate species abundance in the field by directly counting the number of individuals. Therefore, the abundance of bryophyte species is commonly estimated as percent cover or scored as cover class. A number of analyses of the plants occurred in the ground layer, and abundance data are represented in the form of cover class instead of percent cover^52,53^. In our study, we employed 7-level cover classes. The cut-off points for the cover classes were 0, 1%, 5%, 25%, 75%, 95%, and 99%, corresponding to 1–7 cover class codes^54^.

Species richness was represented by the number of species in a subplot, while the importance value and the Shannon-Wiener index were calculated using the following equations^54^, respectively:

1 $$IV=RF+RC$$

where IV is the importance value; RF is the relative frequency, and RC is the relative cover;

2 $$H^{\prime} =-\,\sum _{i=1}^{s}{P}_{i}\,\mathrm{ln}\,{P}_{i}$$

where *H*′ is the Shannon-Wiener index; *s* is the number of species; and *P*_*i*_ is the relative abundance of the *i*-th species, represented by relative cover class.

We calculated two functional diversity metrics, functional dispersion (*FDis*) and Rao’s quadratic entropy (*Rao’s Q*) based on bryophyte traits represented by growth forms, range of distribution, and substrate preference.

Functional dispersion, or *FDis*, is calculated using the following equations^55^:

3 $$FDis=\sum _{i=1}^{s}{a}_{i}{z}_{i}/\sum _{i=1}^{s}{a}_{i}$$

where *a*_*i*_ is the relative cover of the *i*-th species; and *z*_*i*_ is the weighted distance of the *i*-th species to the trait value centroid.

*Rao*’*s Q* is calculated using the following equation^56^:

4 $$Rao^{\prime} sQ=\sum _{i=1}^{s-1}\sum _{j=i+1}^{s}{d}_{ij}{p}_{i}{p}_{i}$$

where *p*_*i*_ is the relative cover; *s* is the number of species; and *d*_*ij*_ is the difference between the *i*-th and the *j*-th species.

### Statistical analysis

We performed two-way cluster analysis on the abundance dataset of bryophyte species composition using UPGMA (unweighted pair group method using arithmetic means) with the Bray-Curtis distance. The two-way cluster analysis simultaneously classifies sample units and gives a graphical representation to observe the ecological similarities or differences of species clusters. Interspecific association can easily be observed from the resulting dendrogram of two-way cluster analysis^54,57^. We assessed the differences in species richness, the Shannon-Wiener index, functional dispersion, and Rao’s quadratic entropy for significance across an edge-to-interior gradient using the Kruskal-Wallis test. The Kruskal-Wallis test is a nonparametric alternative to one-way analysis of variance (ANOVA) and is suitable for the analysis of field ecological data. To evaluate variations in species composition of bryophytes across the edge-to-interior gradient, we performed multi-response permutation procedures (MRPP) on the multivariate dataset of species composition and made pairwise comparison among transects. To assess whether bryophyte species have a specific habitat association and to detect an indicator value of different species for indicating environmental gradient, we performed indicator species analysis (ISA) using Dufrêne and Legendre’s method^54,57^. ISA calculates an indicator value for each species and provides a p-value for each indicator value using permutation.

Two-way cluster analysis, MRPP, ISA, as well as the calculation of community structural attributes and diversity metrics were carried out using PC-ORD 7.0, a software package for multivariate analysis of ecological data (MjM Software, Gleneden Beach, Oregon, USA), while the Kruskal-Wallis test was performed using the software Statistica 8.0 (Statsoft, Inc. Tulsa, OK, USA).

### Data availability

The datasets generated during the current study are available from the corresponding author on reasonable request.

## Electronic supplementary material

Supplementary Table S1

## References

[1] Gensel PG (2008). The earliest land plants. Annu Rev Ecol Evol S.

[2] Hsu CC, Horng FW, Kuo CM (2002). Epiphyte biomass and nutrient capital of a moist subtropical forest in north-eastern Taiwan. J Trop Ecol.

[3] Liu WY, Fox JED, Xu ZF (2002). Nutrient fluxes in bulk precipitation, throughfall and stemflow in montane subtropical moist forest on Ailao Mountains in Yunnan, south-west China. J Trop Ecol.

[4] Song L, Liu WY, Nadkarni NM (2012). Response of non-vascular epiphytes to simulated climate change in a montane moist evergreen broad-leaved forest in southwest China. Biol Conserv.

[5] Loreau M, de Mazancourt C (2013). Biodiversity and ecosystem stability: a synthesis of underlying mechanisms. Ecol Lett.

[6] Ruiz-Benito P (2014). Diversity increases carbon storage and tree productivity in Spanish forests. Global Ecol Biogeogr.

[7] Oliver MJ, Velten J, Mishler BD (2005). Desiccation tolerance in bryophytes: A reflection of the primitive strategy for plant survival in dehydrating habitats?. Integr Comp Biol.

[8] Bengtsson F, Granath G, Rydin H (2016). Photosynthesis, growth, and decay traits in Sphagnum - a multispecies comparison. Ecol Evol.

[9] Degtjarenko P, Marmor L, Randlane T (2016). Changes in bryophyte and lichen communities on Scots pines along an alkaline dust pollution gradient. Environ Sci Pollut R.

[10] Karimi B, Meyer C, Gilbert D, Bernard N (2016). Air pollution below WHO levels decreases by 40% the links of terrestrial microbial networks. Environ Chem Lett.

[11] Rola K, Osyczka P (2014). Cryptogamic community structure as a bioindicator of soil condition along a pollution gradient. Environ Monit Assess.

[12] Wang SQ, Zhang ZH, Wang ZH (2015). Bryophyte communities as biomonitors of environmental factors in the Goujiang karst bauxite, southwestern China. Sci Total Environ.

[13] Mod HK, Heikkinen RK, le Roux PC, Wisz MS, Luoto M (2016). Impact of biotic interactions on biodiversity varies across a landscape. J Biogeogr.

[14] Tessler M, Truhn KM, Bliss-Moreau M, Wehr JD (2014). Diversity and distribution of stream bryophytes: does pH matter?. Freshw Sci.

[15] Cogoni A, Filippino G, Marignani M (2016). Small-scale pattern of bryoflora in Mediterranean temporary ponds: hints for monitoring. Hydrobiologia.

[16] da Costa DP, dos Santos ND, de Rezende MA, Buck WR, Schafer-Verwimp A (2015). Bryoflora of the Itatiaia National Park along an elevation gradient: diversity and conservation. Biodivers Conserv.

[17] Rovere AE, Calabrese GM (2011). Moss diversity in degraded environments under restoring in the Lago Puelo National Park (Chubut, Argentina). Rev Chil Hist Nat.

[18] Liu BY (2016). Physiological responses of two moss species to the combined stress of water deficit and elevated N deposition (II): Carbon and nitrogen metabolism. Ecol Evol.

[19] Song L (2015). Bole bryophyte diversity and distribution patterns along three altitudinal gradients in Yunnan, China. J Veg Sci.

[20] Lin F, Hao Z, Ye J, Jiang P (2006). [Effects of bryophytes in dark coniferous forest of Changbai Mountains on three conifers seed germination and seedling growth]. Ying Yong Sheng Tai Xue Bao.

[21] Alignier A, Deconchat M (2013). Patterns of forest vegetation responses to edge effect as revealed by a continuous approach. Ann Forest Sci.

[22] Craig MD, Stokes VL, Hardy GES, Hobbs RJ (2015). Edge effects across boundaries between natural and restored jarrah (Eucalyptus marginata) forests in south-western Australia. Austral Ecol.

[23] Lippok D (2014). Topography and edge effects are more important than elevation as drivers of vegetation patterns in a neotropical montane forest. J Veg Sci.

[24] Dupuch A, Fortin D (2013). The extent of edge effects increases during post-harvesting forest succession. Biol Conserv.

[25] La Puma IP, Lathrop RG, Keuler NS (2013). A large-scale fire suppression edge-effect on forest composition in the New Jersey Pinelands. Landscape Ecol.

[26] Dawson W, Burslem DFRP, Hulme PE (2015). Consistent Effects of Disturbance and Forest Edges on the Invasion of a Continental Rain Forest by Alien Plants. Biotropica.

[27] Lhotka JM, Stringer JW (2013). Forest edge effects on Quercus reproduction within naturally regenerated mixed broadleaf stands. Can J Forest Res.

[28] Baldwin LK, Bradfield GE (2005). Bryophyte community differences between edge and interior environments in temperate rain-forest fragments of coastal British Columbia. Can J Forest Res.

[29] Dynesius M, Astrom M, Nilsson C (2008). Microclimatic buffering by logging residues and forest edges reduces clear-cutting impacts on forest bryophytes. Appl Veg Sci.

[30] Harper KA (2015). Edge influence on vegetation at natural and anthropogenic edges of boreal forests in Canada and Fennoscandia. J Ecol.

[31] Hofmeister J, Hosek J, Brabec M, Tencik A (2016). Human-sensitive bryophytes retreat into the depth of forest fragments in central European landscape. Eur J Forest Res.

[32] Hylander K (2005). Aspect modifies the magnitude of edge effects on bryophyte growth in boreal forests. J Appl Ecol.

[33] Lobel S, Snall T, Rydin H (2012). Epiphytic bryophytes near forest edges and on retention trees: reduced growth and reproduction especially in old-growth-forest indicator species. J Appl Ecol.

[34] Roberge JM, Bengtsson SBK, Wulff S, Snall T (2011). Edge creation and tree dieback influence the patch-tracking metapopulation dynamics of a red-listed epiphytic bryophyte. J Appl Ecol.

[35] Stewart KJ, Mallik AU (2006). Bryophyte responses to microclimatic edge effects across riparian buffers. Ecol Appl.

[36] Bunker DE, Naeem S (2006). Species diversity and ecosystem functioning. Science.

[37] Cardinale BJ, Palmer MA, Collins SL (2002). Species diversity enhances ecosystem functioning through interspecific facilitation. Nature.

[38] Sanderson MA (2007). Plant species diversity, ecosystem function, and pasture management - A perspective. Can J Plant Sci.

[39] Busby JR, Bliss LC, Hamilton CD (1978). Microclimate Control of Growth Rates and Habitats of the Boreal Forest Mosses, Tomenthypnum nitens and Hylocomium splendens. Ecological Monographs.

[40] Chen J, Franklin JF, Spies TA (1993). Contrasting microclimates among clearcut, edge, and interior of old-growth Douglas-fir forest. Agricultural & Forest Meteorology.

[41] Esseen PA (2006). Edge influence on the old-growth forest indicator lichen Alectoria sarmentosa in natural ecotones. J Veg Sci.

[42] Wicklein HF, Christopher D, Carter ME, Smith BH (2012). Edge Effects on Sapling Characteristics and Microclimate in a Small Temperate Deciduous Forest Fragment. Natural Areas Journal.

[43] Haghroosta T, Wan RI (2017). Typhoon activity and some important parameters in the South China Sea. Weather & Climate Extremes.

[44] Liu B, Pan L (2012). A review of the effect of typhoon on forests. Acta Ecologica Sinica.

[45] Hock Z, Szovenyi P, Schneller JJ, Toth Z, Urmi E (2008). Bryophyte diaspore bank: A genetic memory? Genetic structure and genetic diversity of surface populations and diaspore bank in the liverwort Mannia fragrans (Aytoniaceae). Am J Bot.

[46] Miller NG, McDaniel SF (2004). Bryophyte dispersal inferred from colonization of an introduced substratum on Whiteface Mountain, New York. Am J Bot.

[47] Schmalholz M, Hylander K (2011). Boulders increase resistance to clear-cut logging but not subsequent recolonization rates of boreal bryophytes. Oecologia.

[48] Hu YQ, Su ZY, Li WB, Li JP, Ke XD (2015). Influence of tree species composition and community structure on carbon density in a subtropical forest. Plos One.

[49] Jia, Z., Wu, L., Wang, T. & Wen, Z. Relationship between autumn rainfall anomalies in South China and typhoons activity,and the anomalous sea surface temperature characteristics. *Acta Oceanologica Sinica* (2015).

[50] He, S. Y. *et al*. Topography-associated thermal gradient predicts warming effects on woody plant structural diversity in a subtropical forest. *Sci Rep-Uk* **7**, 10.1038/srep40387 (2017). 10.1038/srep40387 PMC5220297 28067326

[51] Wu, D. & Zhang, L. *The Bryophyte Flora of Guangdong*. (Guangdong Science and Technology Press, 2013).

[52] Mboukou-Kimbatsa I, Bernhard-Reversat F, Loumeto JJ, Ngao J, Lavelle P (2007). Understory vegetation, soil structure and soil invertebrates in Congolese eucalypt plantations, with special reference to the invasive plant Chromolaena odorata and earthworm populations. Eur J Soil Biol.

[53] Nelson PR, McCune B, Swanson DK (2015). Lichen traits and species as indicators of vegetation and environment. Bryologist.

[54] McCune, B., Grace, J. B. & Urban, D. L. *Analysis of Ecological Communities*. (MjM Software Design, 2002).

[55] Laliberte E, Legendre P (2010). A distance-based framework for measuring functional diversity from multiple traits. Ecology.

[56] Botta-Dukat Z (2005). Rao’s quadratic entropy as a measure of functional diversity based on multiple traits. J Veg Sci.

[57] Peck, J. E. *Multivariate Analysis for Community Ecologists: Step-by-Step Using PC-ORD*. (MjM Software Design, 2010).

