# Supplementary material for: Species composition and diversity of ground bryophytes across a forest edge-to-interior gradient
Source: Sci Rep. 2018 Aug 8;8:11868. doi: 10.1038/s41598-018-30400-1 (PMC6082881; doi:10.1038/s41598-018-30400-1)
Supplement: Supplementary file 1 — Supplementary Table S1 [file 41598_2018_30400_MOESM1_ESM.pdf]

Species composition and diversity of ground bryophytes across a forest edge-to-interior gradient

Tiantian Jiang, Xuecheng Yang, Yonglin Zhong, Qiming Tang, Ying Liu & Zhiyao Su*
College of Forestry and Landscape Architecture, South China Agricultural University, Guangzhou 510642, China

***Corresponding author:**
Z.S. (Zhiyao Su): zysu@scau.edu.cn

Supplementary information

Supplementary Table S1: Bryophyte cover-class dataset.

Table S1. Bryophyte cover-class dataset. For Species ID and the corresponding species code and species name, see explanations under this table.

Plot	Species ID																																			
	1	2	3	4	5	6	7	8	9	10	11	12	13	14	15	16	17	18	19	20	21	22	23	24	25	26	27	28	29	30	31	32	33	34	35	36	37
P01	2	0	2	0	2	0	0	1.5	0	1	0	2	2	2	0	0	0	0	0	1	0	0	0	1	0	0	0	0	0	2	0	0	0	0	0	2	0
P02	0	0	0	0	2	1	0	0	0	0	0	1	0	0	0	0	0	0	0	0	0	0	0	0	0	1	0	0	0	2	0	0	0	0	0	1	0
P03	0	0	0	0	2.5	0	0	0	0	0	0	0	0	0	0	0	0	0	0	0	0	0	0	0	0	0	0	0	0	2	0	0	0	0	0	0	0
P04	0	0	0	0	2	0	0	0	0	0	0	0	0	0	0	0	0	0	0	0	0	0	0	0	0	0	0	0	0	2	0	0	0	0	0	0	0
P05	0	0	0	0	0	0	0	0	0	0	0	0	0	0	0	0	0	0	0	0	0	0	0	0	0	0	0	0	0	2	0	0	0	0	0	3	0
P06	0	2	1	0	2	0	0	0	0	0	0	0	0	0	0	0	0	0	0	2	0	2	0	0	0	0	0	0	0	3	0	0	0	0	0	0	3
P07	0	3	0	0	2	0	0	0	0	2	0	0	0	0	0	0	0	0	0	2.5	0	2	0	0	0	0	0	0	0	2	0	0	0	0	0	0	0
P08	0	0	0	0	0	0	0	0	0	1	0	0	0	0	0	0	0	0	0	2	0	0	0	0	0	0	0	0	0	2	0	0	0	0	0	0	0
P09	0	1	0	0	0	0	0	0	0	1	0	0	0	0	0	0	0	0	0	1.5	0	0	0	0	0	0	0	0	0	2	0	0	0	0	0	0	0
P10	0	0	0	0	1	0	0	0	0	1	0	0	0	0	0	0	0	0	0	2	0	0	0	0	0	0	0	0	0	0	0	0	0	0	0	0	0
P11	0	0	0	0	1	0	3	0	0	1.5	0	0	0	0	0	0	0	0	0	2	2	0	0	0	0	0	0	0	0	2	0	0	0	0	0	0	0
P12	0	0	0	0	2	0	0	0	0	2	0	0	0	0	0	0	0	0	0	0	0	0	0	0	0	0	0	0	0	2	0	0	0	0	0	0	0
P13	0	2	0	0	2	0	0	0	0	0	0	0	0	0	0	0	0	0	0	0	0	0	0	0	0	0	0	0	0	2	0	0	0	0	0	4	0
P14	0	0	0	0	2	0	0	0	0	0	0	0	0	0	0	0	0	0	0	0	0	0	0	0	0	0	0	0	0	2.5	0	0	0	0	0	0	0
P15	0	0	0	0	2	0	0	0	0	0	0	0	0	0	0	0	0	0	0	0	0	0	0	0	0	0	0	0	0	2	0	0	0	0	0	0	0
P16	0	2	0	0	2	0	0	0	0	0	0	0	0	0	0	0	0	0	0	2	0	5	0	0	0	0	0	0	0	2	0	0	0	0	0	0	0
P17	0	2	0	0	0	0	0	0	0	1	0	0	0	0	0	0	0	0	0	3	0	1	0	0	0	0	0	0	0	2	0	0	0	0	2	0	0
P18	0	2.5	0	0	2.5	0	0	0	0	2	0	0	0	0	0	0	0	0	0	2	0	0	0	0	0	0	0	0	0	2	0	0	0	0	0	0	2
P19	0	2	0	0	0	0	0	0	0	2	0	0	0	0	0	0	0	0	0	3	0	0	0	0	0	0	0	0	0	2	0	0	0	0	0	0	0
P20	0	0	0	0	0	0	0	0	0	3	0	0	0	0	0	0	0	0	0	2	0	2	0	0	0	0	0	0	0	2	0	0	0	0	0	0	0
P21	0	0	0	0	3	0	0	0	0	2	0	0	0	0	0	0	0	0	0	0	1	0	0	0	0	0	0	0	0	1	0	0	0	0	0	0	0
P22	0	0	0	0	1.5	0	0	0	0	2	0	0	0	0	0	0	0	0	0	0	0	0	0	0	0	0	0	0	0	2	0	0	0	0	0	0	0
P23	0	0	0	0	2	0	0	0	0	0	0	0	4	0	0	0	0	2	0	0	0	0	0	3	0	0	2.5	0	2	2	0	0	0	0	0	0	2
P24	0	0	1	0	2	0	0	0	0	0	0	2	0	0	0	0	0	0	0	0	0	0	0	0	0	0	0	0	0	2	0	0	0	0	0	0	0
P25	0	3	0	0	3	0	0	0	0	3	0	0	0	0	0	0	0	0	0	2	2	3.5	0	0	0	0	0	0	0	2	0	0	0	0	0	0	0

P26	0	2.5	0	0	2.5	0	0	0	0	0	0	0	0	0	0	0	0	0	0	3	0	2	0	0	0	0	0	0	0	2	0	0	0	0	0	0	0
P27	0	2.5	2	0	2	0	0	0	0	2	0	2	0	0	0	0	0	0	3	2	0	2	0	0	0	0	0	0	0	2	0	0	0	0	2	0	0
P28	0	3	0	0	0	0	0	0	0	1.5	0	0	0	0	0	0	0	0	0	2	0	2	0	0	0	0	0	0	0	2	0	0	0	0	0	0	0
P29	0	2	0	0	0	0	0	0	0	2	0	0	0	0	0	0	0	0	0	2	0	1	0	0	0	0	0	0	0	2	0	0	0	0	0	0	0
P30	0	2	0	0	0	0	0	0	0	0	0	0	0	0	0	0	0	0	3	2	0	0	0	0	0	0	0	0	0	2	0	0	0	0	0	0	0
P31	0	2	0	0	1	0	2	1	0	0	0	2	1.5	0	0	0	2	1.5	0	1	1	0	0	0	0	0	0	0	0	2.5	3	2	0	2	0	0	1
P32	0	0	0	0	0	0	0	0	2	0	0	5	3	4	0	2	0	3	0	0	0	0	0	0	0	0	3.5	3	0	0	4	0	0	0	0	0	3
P33	0	0	0	0	0	0	2	3.5	0	0	0	0	4	0	4	0	2	3	0	0	0	0	0	0	0	0	3.5	0	0	3	3	4	0	3	0	0	3
P34	0	0	0	0	0	0	2	0	0	0	3	0	3	0	4	0	0	2	0	2	1	4	0	0	0	0	3	0	0	3	0	3	0	2	0	0	3
P35	0	0	0	0	2	0	0	0	0	3	0	0	0	0	0	0	0	0	0	2	0	0	0	0	0	0	0	0	0	2	0	0	0	0	0	0	1
P36	0	2	2	0	3	0	0	0	0	0	0	0	0	0	0	0	0	0	3	2	0	0	0	0	0	0	0	0	0	3	0	0	0	0	0	0	0
P37	0	0	2	2	0	0	0	0	0	0	0	1	0	0	0	0	0	0	0	2	0	3	2	0	0	0	0	0	0	2	0	0	2.5	0	0	0	0
P38	0	2	0	0	0	0	0	0	0	0	0	0	0	0	0	0	0	0	0	3.5	0	0	0	0	0	0	0	0	0	2	0	0	0	0	0	0	0
P39	0	0	0	0	2	0	0	0	0	2	0	0	0	0	0	0	0	0	0	3	0	2	0	0	0	0	0	0	0	2	0	0	0	0	0	0	0
P40	0	3	0	0	0	0	0	0	0	0	0	0	0	0	0	0	0	0	0	2	0	0	0	0	0	0	0	0	0	2	0	0	0	0	0	0	0
P41	0	2	0	0	2.5	0	0	0	0	0	0	0	0	0	0	0	0	0	0	2.5	0	0	0	0	3	0	0	0	0	3	0	0	0	0	0	0	0
P42	2	0	0	0	1	0	0	0	0	0	0	0	0	0	0	0	0	0	0	3	0	0	0	0	0	0	0	0	0	2	0	0	0	0	0	0	3
P43	3	0	3	0	2	0	4	2.5	0	2	1.5	2	3	3	2	0	2	3	0	0	3	0	0	2	0	0	0	0	0	2	0	0	3.5	0	0	2	3
P44	3	0	2	0	0	0	0	2	0	0	0	0	3.5	4	0	0	0	2	0	0	3	2	0	2	0	0	0	0	0	0	0	0	0	0	0	0	3
P45	2	0	2.5	0	0	0	3	0	0	2	0	0	3	3	0	0	0	2	0	0	3	0	0	3	0	0	0	0	0	2	0	0	0	2	0	0	4
P46	0	3.5	0	0	0	0	0	0	0	2	0	0	0	0	0	0	0	0	0	4	0	0	0	0	0	0	0	0	0	3	0	0	0	0	0	0	0
P47	0	3	0	0	0	0	0	0	0	3	0	0	0	0	0	0	0	0	0	4	0	0	0	0	0	0	0	0	0	4	0	0	0	0	0	0	0
P48	0	3	0	0	0	0	0	0	0	0	0	0	0	0	0	0	0	0	0	2	0	0	0	0	0	0	0	0	0	2	0	0	0	0	0	0	0
P49	0	0	0	0	0	0	0	0	0	2	0	0	0	0	0	0	0	0	0	2.5	0	0	0	0	0	0	0	0	0	2.5	0	0	0	0	2.5	0	0
P50	0	3	2	0	0	0	0	0	0	2	0	0	0	0	0	0	0	0	0	2	0	0	0	0	0	0	0	0	0	3	0	0	0	0	0	0	0

Explanations for species ID (SpID), species code (SpCode) and species name

SpID	SpCode	Species
1	AERWAL	*Aerobryopsis wallichii*
2	BAZTRI	*Bazzania tridens*
3	BROHEN	*Brotherella henonii var. henonii*
4	CAMATR	*Campylopus atrovirens*
5	CHILAT	*Chiloscyphus latifolius*
6	CHIPRO	*Chiloscyphus profundus*
7	ECTDEA	*Ectropothecium dealbatum*
8	ENTSCH	*Entodon schleicheri*
9	FAUTEN	*Fauriella tenuis*
10	FISLAX	*Fissidens laxus*
11	FISNOB	*Fissidens nobilis*
12	FISOBL	*Fissidens oblongifolius*
13	HAPMIC	*Haplocladium microphyllum*
14	HERTOC	*Herpetineuron toccoae*
15	HETZOL	*Heteroscyphus zolliingeri*
16	HOMFLA	*Homaliodendron flabellatum*
17	HOMTRI	*Homalia trichomanoides*
18	HOMTRJ	*Homalia trichomanoides var. japonica*
19	HYPFAU	*Hypnum fauriei*
20	KURGON	*Kurzia gonyotricha*
21	LEJEIF	*Lejeunea eifrigii*
22	LEUJUN	*Leucobryum juniperoideum*
23	MACSCH	*Macromitrium schmidii* var. *Macroperichaetialium*
24	METCON	*Metzgeria conjugata*
25	NECCAL	*Neckeropsis calcicola*
26	PALLYE	*Pallavicinia lyellii*
27	PLAFLE	*Plagiochila flexuosa*
28	PLARHY	*Plagiomnium rhynchophorum*
29	POGINF	*Pogonatum inflexum*
30	PSEPOH	*Pseudotaxiphyllum pohliaecarpum*
31	RADOBS	*Radula obscura*
32	RHYPAL	*Rhynchostegium pallidifolium*
33	SEMSUB	*Sematophyllum subpinnatum*
34	SPRPOL	*Spruceanthus polymorphus*
35	SYRPRO	*Syrrhopodon prolifer*
36	TAXTAX	*Taxiphyllum taxirameum*
37	THUPRI	*Thuidium pristocalyx*
